# Anti-hyperalgesic and anti-inflammatory effects of 4R-tobacco cembranoid in a mouse model of inflammatory pain

**DOI:** 10.1186/s12950-023-00373-8

**Published:** 2024-01-24

**Authors:** Luis G. Rivera-García, Adela M. Francis-Malavé, Zachary W. Castillo, Calvin D. Uong, Torri D. Wilson, P. A. Ferchmin, Vesna Eterovic, Michael D. Burton, Yarimar Carrasquillo

**Affiliations:** 1 Division of Intramural Research National Center for Complementary and Integrative Health, 35 Convent Drive, Building 35A / Room 1E-410, Bethesda, MD 20892 USA; 2 Department of Neuroscience, Universidad Central Del Caribe School of Medicine, Bayamon, Puerto Rico USA; 3 Neuroimmunology and Behavior Group, Department of Neuroscience, Center for Advanced Pain Studies (CAPS), School of Behavioral and Brain Sciences, University of Texas, Dallas, USA; 4 National Institute On Drug Abuse, National Institutes of Health, 35 Convent Drive, Building 35A / Room 1E-410, Bethesda, MD 20892 USA

**Keywords:** Inflammatory pain, Tobacco cembranoid, 4R, Hyperalgesia, Paw edema, α7 nicotinic acetylcholine receptors, Persistent analgesia, Macrophage

## Abstract

4R is a tobacco cembranoid that binds to and modulates cholinergic receptors and exhibits neuroprotective and anti-inflammatory activity. Given the established function of the cholinergic system in pain and inflammation, we propose that 4R is also analgesic. Here, we tested the hypothesis that systemic 4R treatment decreases pain-related behaviors and peripheral inflammation via modulation of the alpha 7 nicotinic acetylcholine receptors (α7 nAChRs) in a mouse model of inflammatory pain. We elicited inflammation by injecting Complete Freund’s Adjuvant (CFA) into the hind paw of male and female mice. We then assessed inflammation-induced hypersensitivity to cold, heat, and tactile stimulation using the Acetone, Hargreaves, and von Frey tests, respectively, before and at different time points (2.5 h – 8d) after a single systemic 4R (or vehicle) administration. We evaluated the contribution of α7 nAChRs 4R-mediated analgesia by pre-treating mice with a selective antagonist of α7 nAChRs followed by 4R (or vehicle) administration prior to behavioral tests. We assessed CFA-induced paw edema and inflammation by measuring paw thickness and quantifying immune cell infiltration in the injected hind paw using hematoxylin and eosin staining. Lastly, we performed immunohistochemical and flow cytometric analyses of paw skin in α7 nAChR-cre::Ai9 mice to measure the expression of α7 nAChRs on immune subsets. Our experiments show that systemic administration of 4R decreases inflammation-induced peripheral hypersensitivity in male and female mice and inflammation-induced paw edema in male but not female mice. Notably, 4R-mediated analgesia and anti-inflammatory effects lasted up to 8d after a single systemic administration on day 1. Pretreatment with an α7 nAChR-selective antagonist prevented 4R-mediated analgesia and anti-inflammatory effects, demonstrating that 4R effects are via modulation of α7 nAChRs. We further show that a subset of immune cells in the hind paw expresses α7 nAChRs. However, the number of α7 nAChR-expressing immune cells is unaltered by CFA or 4R treatment, suggesting that 4R effects are independent of α7 nAChR-expressing immune cells. Together, our findings identify a novel function of the 4R tobacco cembranoid as an analgesic agent in both male and female mice that reduces peripheral inflammation in a sex-dependent manner, further supporting the pharmacological targeting of the cholinergic system for pain treatment.

**Supplementary Information:**

The online version contains supplementary material available at 10.1186/s12950-023-00373-8.

## Background

Nicotiana plants, commonly referred to as tobacco plants, were used by many cultures in the past for a wide range of medicinal purposes, such as wound and burn healing, pain relief, and as a therapeutic option for different diseases [1–3]. Nicotine is the best-known alkaloid of the tobacco plant and is known to bind to all nicotinic cholinergic receptors [4]. Endogenously, nicotinic acetylcholine receptors (nAChRs) can be activated by acetylcholine and have been implicated in various physiological functions such as cognition, inflammation, and pain processing [5, 6]. nAChRs are ligand-gated ion channels found throughout the nervous system and are also found in subsets of immune cells [7–9]. Preclinical studies have shown the therapeutic potential of targeting nAChRs for their anti-inflammatory effects via their modulation of the innate immune response [8].

Consistent with the proposed function of the cholinergic system in pain processing and inflammation, previous studies in rodents have shown that a basal cholinergic tone modulates nociceptive processing. Specifically, the cholinergic tone is increased following injury, and manipulation of this system by endogenous or exogenous ligands can have analgesic and anti-inflammatory effects [10–12]. Given the highly addictive and harmful properties of nicotine, however, efforts have been made to identify pharmacological alternatives to modulate the cholinergic system to treat pain and inflammation [6, 13].

**Fig. 1:**
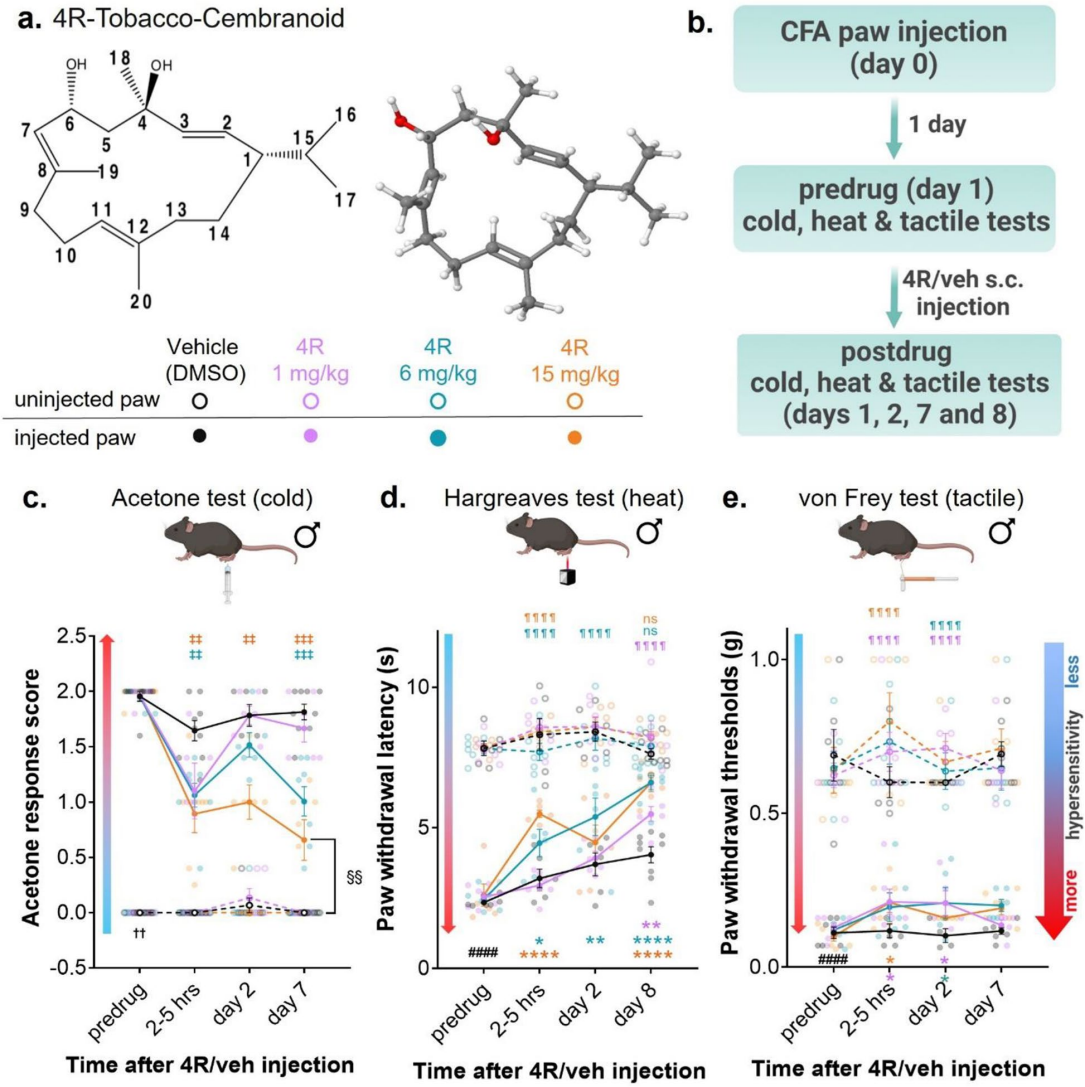
Systemic administration of 4R tobacco cembranoid reduces inflammation-induced peripheral hypersensitivity (**a**) Chemical structure (left panel) and 3D molecular model (right panel) of 4R-tobacco cembranoid. Oxygen, hydrogen, and carbon atoms are colored red, white, and grey, respectively. (**b**) Experimental timeline for acetone (cold), Hargreaves (heat) and von Frey (tactile) tests in CFA-injected and non-injected hind paws before and at different times after 4R (1, 6 and 15 mg/kg) or vehicle administration. (**c**) Acetone response score, (**d**) withdrawal latency to heat stimulation (**e**) withdrawal threshold to tactile stimulation. All values are expressed as mean ± SEM. *n* = 4–9 animals per treatment and test. Two-way ANOVA followed by posthoc Tukey’s multiple comparisons test: CFA-injected paw vs uninjected paw in pre-drug vehicle (veh) conditions, *p* < 0.0001 (####), 4R treated CFA-injected paw vs vehicle uninjected paw, *p* < 0.0001 (¶ ¶ ¶ ¶); not significant = ns. Two-way ANOVA followed by post hoc Dunnett’s multiple comparisons test: CFA-injected paws in vehicle vs 4R systemic treatment of the same time-point, *p* < 0.05 (*), *p* < 0.01 (**), *p* < 0.001 (***), *p* < 0.0001 (****). Wilcoxon matched-pairs signed rank test: CFA-injected paw vs uninjected paw in pre-drug vehicle (veh) conditions, *p* < 0.01 (††), Non-parametric Mann–Whitney multiple comparisons test followed by Holm-Šídák method; CFA-injected paws in vehicle vs 4R systemic treatment of the same time-point, adjusted *p* < 0.01 (‡‡), adjusted *p* < 0.001 (‡‡‡); vehicle (veh) uninjected paw (day 7) vs 4R 15 mg/kg CFA-injected paw (day 7), adjusted *p* > 0.05 (§§). Results of multiple comparisons between vehicle (veh) and 4R 1 mg/kg, 6 mg/kg or 15 mg/kg are shown in purple, teal, and orange, respectively

In addition to nicotine, tobacco plants contain many other chemical compounds, including cyclic diterpenoids called cembranoids found in tobacco leaves, flowers, and smoke [14, 15]. The first identified tobacco cembranoids were isolated by Roberts and Rowland in 1962 [16]. Since then, at least 89 other tobacco cembranoids have been described [17]. Whether tobacco cembranoids contributed to the medicinal properties attributed to the tobacco plant in the past, including wound healing and pain relief, has not been established. In the present study, we began to address this question by evaluating the potential analgesic and anti-inflammatory effects of the tobacco cembranoid (1S, 2E, 4R, 6R, 7E, 11E)-cembra-2,7,11-triene-4,6-diol, abbreviated as 4R. 4R was initially extracted and purified from tobacco leaves, has the molecular formula C_20_H_34_O_2_, and the chemical structure is shown in Fig. 1. We selected 4R for this study because it is one of the best functionally characterized tobacco cembranoids [18–22]. Importantly, 4R has been previously shown to have anti-inflammatory and neuroprotective effects and to inhibit prostaglandin synthesis [20, 23–27].

Additional studies have further shown that 4R binds to nAChRs and inhibits three human nAChRs subtypes: neuronal α4β2 (IC50 = 19.1 µM) and α3β4 (IC50 = 2.2 µM) and the embryonic muscle AChR α1β1γδ-AChR (IC50 = 6.6 µM), demonstrating that 4R is a nicotinic receptor ligand [15, 28]. Among the different types of nAChRs, the α7 nAChR has received increasing attention as a potential therapeutic target for chronic inflammation and neuropathic pain [29–31]. Consistent with this, α7 nAChRs have been shown to be expressed in most types of immune cells, including macrophages, T-cells, B-cells, mast cells, and neutrophils [31–34]. Studies have further shown that pharmacological activation of α7 nAChRs reduces inflammation and that deficiencies in α7 nAChRs exacerbate inflammation [32, 35–38]. At the behavioral level, α7 nAChR agonists have been shown to ameliorate pain in various animal pain models [31, 33, 39, 40]. Together these studies strongly position α7 nAChRs as a critical determinant of inflammation and pain-related behaviors.

Based on these combined findings and the proposed function of the cholinergic system in pain modulation and inflammation, we hypothesized that 4R could also decrease injury-induced hypersensitivity in a mouse model of inflammatory pain via modulation of α7 nAChRs. To test these hypotheses, we employed multidisciplinary approaches including pharmacology, behavioral pain assays, genetically modified mice, histology, and flow cytometry. Our pharmacological experiments demonstrate that inflammation-induced peripheral hypersensitivity is reduced with systemic 4R treatment in both male and female mice. Measurements of paw edema and histological assessment of paw samples further show that 4R reduces peripheral inflammation in a sex-dependent manner. Additionally, using Methyllycaconitine (MLA), a selective antagonist of α7 nAChRs, we demonstrate that the anti-hyperalgesic and anti-inflammatory effects of 4R are mediated through α7 nAChRs. Flow cytometry and immunohistochemical analyses of paw samples from *Chrna7*-Cre::Ai9 mice, which are genetically modified to express tdTomato in α7 nAChRs + cells, further indicate that a subset of macrophages and T-cells expresses the α7 nAChRs. However, no measurable changes in the number of α7 nAChR-expressing immune cells were observed following inflammation. Together, our results identified a novel analgesic function for 4R and further support the use of pharmacological agents targeting the cholinergic system to treat pain and inflammation.

## Materials and methods

### Mice

Male and female C57BL/6 J (Jackson Labs; stock no. 00064) mice were used for all the behavioral experiments. To visualize and detect cells expressing α7 nAChRs in histological and flow cytometric experiments, we used the genetically modified mice described below. *Chrna7-*cre heterozygous male and female mice, stock Tg(Chrna7-cre)NP348Gsat/Mmucd, RRID:MMRRC_034694-UCD, were obtained from the Mutant Mouse Resource and Research Center (MMRRC) at University of California at Davis, an NIH-funded strain repository, and was donated to the MMRRC by Nathaniel Heintz, Ph.D., The Rockefeller University, GENSAT and Charles Gerfen, Ph.D., National Institutes of Health, National Institute of Mental Health. *Chrna7-*cre heterozygous male and female mice were backcrossed to C57BL/6NJ (Jackson Labs; stock no. 005304) for ten generations. *Chrna7-*cre heterozygous male and female mice in C56BL/6NJ background were then crossed with homozygous Ai9 (The Jackson Laboratory, stock no. 007909). The offspring, referred to in this study as α7^tdTomato^, were used for immunohistochemistry (IHC) and flow cytometry experiments. The genotype of offspring mice was assessed for the presence of Cre-recombinase using DNA from tail biopsies and polymerase chain reaction (PCR, Transnetyx). The following primer sequences were used: TTAATCCATATTGGCAGAACGAAAACG (forward) and CAGGCTAAGTGCCTTCTCTACA (reverse).

Animals were housed in groups of 3–5 littermates in a temperature-controlled facility, with ad libitum access to food and water under reversed 12 h/12 h light/dark cycle (9 pm to 9 am light). All behavioral experiments were performed during the dark phase under red light. For behavior experiments, one week prior to experiments, mice were transferred in pairs to a new home cage divided with perforated plexiglass, with one mouse in each compartment. Mice were handled daily for five days before behavioral testing, as previously described [41]. Procedures performed with mice were approved by the Animal Care and Use Committee of the National Institute of Neurologic Disorders and Stroke, the National Institute of Deafness and Other Communication Disorders, and the University of Texas at Dallas Institutional Animal Care and Use Committee per the National Institutes of Health (NIH) guidelines. All behavioral experiments were performed by an investigator blind to experimental treatments. Pairs of animals within a home cage were randomly assigned to individual experimental groups. After treatment, mice were returned to their original home cage. This randomization system allowed mice with the same treatment to be housed together. Male and female mice were never tested simultaneously in the same behavior room. All experimental procedures were replicated at least two times. For the behavioral experiments, the minimum sample size per sex was determined by a power calculation using G*power (version 3.1.9.2) with effect size (f) set to 0.75, at 95% power with a set to 0.05 [42–44].

### CFA model of inflammatory pain and behavioral tests

Mice were lightly anesthetized with isoflurane (0.5%–1% at a flow rate of 0.5 L/min), and 10 µL of Complete Freund’s Adjuvant (CFA, F5881, Sigma Aldrich) was injected subcutaneously into the plantar area of the right hind paw using an insulin syringe (Sure Comfort 29 g × 1/2″ 3/10 cc). Day 0 is defined as the day of CFA injection. Drug solutions used for systemic injections (vehicle or 4R) were made fresh on the day of administration, and a single subcutaneous (s.c.) injection of 56—70 µL was administered into the loose skin over the shoulder on day 1. CFA-induced inflammation and hyperalgesia were assessed on day 1 before s.c. injection of vehicle or 4R (1, 6, or 15 mg/kg body weight). The doses of 4R were selected based on previous pharmacokinetic studies in rats [45] and extrapolated to mice to account for species differences as previously described [46]. The effects of s.c. treatment with 4R or vehicle on CFA-induced inflammation and hyperalgesia were assessed on days 1, 2, 7, and 8 after the injections. Day 1 effects were measured 2–5 h after s.c. injections.

To evaluate the contribution of α7 nAChRs, we used the α7 nAChRs selective antagonist, Methyllycaconitine (MLA). For these experiments, mice received a single vehicle or MLA (10 mg/kg body weight) injection subcutaneously 15 min before the injection of vehicle or 4R (15 mg/kg body weight). This dose of MLA has been shown to effectively block the anti-nociceptive effects of pharmacological treatments targeting α7 nAChRs [47, 48]. The rest of the experiments were performed identically to the 4R experiments described above.

The dorsal–ventral diameter of the CFA-treated and untreated hind paws were measured as an indirect measurement of inflammation using a microcaliper (UX-97152–17, Cole Palmer). For all behavioral tests, animals were individually placed inside a custom-made white plexiglass testing chamber (11 × 11x13 cm) on an elevated mesh (for acetone and von Frey tests) (NIH Section on Instrumentation) or a glass platform heated to 30ºC (for the heat test).

### von Frey

Withdrawal thresholds to tactile stimulation, defined as paw withdrawal followed by a brief shake or lick of the paw, were measured using graded monofilaments (North Coast Medical, Inc. San Jose, CA) after animals were habituated to the testing chamber and room for 3 h, as previously described [49]. Starting with the smallest filament, the tip was pressed against the plantar area of the hind paw until it bent at 30º for approximately one second. The procedure was repeated five times per filament. The filament that elicited paw withdrawal at least three out of five times was recorded as the mechanical threshold for that trial. The average of three trials was considered the mechanical threshold of the paw tested.

### Acetone test

Cold sensitivity was assessed using the acetone evaporative test [50], adapted to test mice. An acetone drop was briefly applied to the plantar region of the hind paw and nocifensive responses were scored using a modified scoring system previously described [49, 51]. The scoring system ranges from 0 to 2, indicating the following: 0 = no reaction or an immediate transient lifting or shaking of the hind paw that subsides immediately, 1 = lifting, licking, and/or shaking of the hind paw, which continues beyond the initial application, but subsides within 5 s, and 2 = protracted, repeated lifting, licking, and/or shaking of the hind paw. The response to acetone application was observed for approximately one minute and scored. The average of three stimulations per hind paw represents the score of the paw tested.

### Hargreaves test

The Hargreaves test was used to assess sensitivity to heat, as previously reported [49]. Animals were habituated for one hour in ventilated testing chambers placed on a heated glass surface (30ºC). Paw withdrawal latencies were measured after stimulating the hind paw with a heat light aimed at the center of the plantar surface using an active intensity of 25 (intensity of light source defined by the manufacturer; IITC Life Science, Woodland Hills, CA). Three to five stimulations per hind paw were logged, and the average was reported.

### Drugs

The tobacco cembranoid (1S,2E,4R,6R,7E,11E)-2,7,11-cembratriene-4,6-diol (4R) was obtained from El Sayed Research Foundation; University of Louisiana–Monroe, College of Pharmacy and prepared as previously described [52]. The purity of the batch used for these experiments was more than 98%, determined by Nuclear Magnetic Resonance (NMR) spectroscopy and thin layer chromatography (TLC). 4R was dissolved in dimethyl sulfoxide (DMSO) (D2650; Sigma Aldrich) to make a stock solution with a final concentration of 20 mM. The selective antagonist MLA (1029; Tocris) was dissolved in saline to make a 10 mM stock solution. A 2.5 mM 4R solution was made from the 4R (20 mM) stock solution to produce the lowest 4R dose injected (1 mg/kg body weight). In all experiments, mice were tested in cohorts of 6 and always included at least one animal per dose. Individual doses per animal were calculated based on body weight. All syringes in a cohort were brought to the same final volume with DMSO as part of the blinding process for testing. The range of injected volumes used was 56 – 70 µL.

### Samples origin for histology

Mice were injected with CFA on the right hind paw seven days before collecting the samples. 24 h after CFA injection, mice were treated with 4R (15 mg/kg) or vehicle (DMSO) by a single s.c. injection. On day eight after the CFA injection, mice were anesthetized with 1.25% Avertin (2,2,2-tribromoethanol and tert-amyl alcohol in 0.9% NaCl; 0.025 ml/g body weight) followed by transcardial perfusion with 37 °C 0.9% NaCl to clear the blood and 100 ml of ice-cold 4% paraformaldehyde (Sigma, 158127) made in 1 × phosphate-buffered saline (PBS) (pH = 7.4 ± 0.05) (4% PFA/PB). After transcardial perfusion, the left (uninjected) and right (injected) hind paws were removed by cutting the tibia and fibula halfway between the knee and ankle joints. Collected samples were post-fixed with 4% PFA/PB overnight at 4ºC and sent to Histoserv Inc. (Germantown, MD) the next day for histological processing. After mild decalcification for 3.5 weeks, hind paws were embedded in paraffin blocks and a total of three 10-µm cross-sectional cuts of the foot pad were collected per hind paw at the site of intraplantar injection and fixed to glass slides for subsequent staining. Two of these sections were collected 20 µm apart and the third one was collected 100 µm apart.

### H&E staining and Iba1 immunofluorescence

One of the collected cross-sectioned cuts of the hind paws foot pad was stained with hematoxylin and eosin (H&E) by Histoserv (Germantown, MD). The other two sections, separated by 100 µm, were used for immunostaining Histoserv (Germantown, MD). Slides were air-dried, fixed for 2 min in neutral buffered formalin with methanol, and air-dried again. 5% normal goat serum (NGS) with 0.5% bovine serum albumin (BSA) and 0.1% Triton X-100 was used for blocking solution. Sections were incubated overnight at 4 °C in rabbit anti-ionized calcium-binding adaptor molecule 1 (Iba1) (1:2000; 019–19741; Fujifilm Wako Pure Chemical Corporation) diluted in 1.5% NGS, 0.5% BSA, and 0.1% Triton X-100. Sections then were incubated in secondary antibody tagged with fluorophore Alexa Fluor 647-conjugated goat anti-rabbit (1:100, Invitrogen, A21244). Finally, slides were wet mounted using Prolong Gold with DAPI (P36931, Invitrogen) and a coverslip, air dried, and the edges were sealed.

### Tissue collection and cell isolation for flow cytometry

On day 8, mice were deeply anesthetized using isoflurane and euthanized via decapitation. Ipsi- and contralateral hind paws were collected for flow cytometry. The whole foot was removed at the ankle joint, and plantar skin (including the subdermal layer) from the foot pad was dissected into ice-cold sterile 1 × DPBS (Hyclone, SH30028) before downstream processing. Fresh skin samples were centrifuged at 400 × g for 3 min, and supernatants were removed and treated with a mix of Collagenase A (Sigma, 10103586001) and Collagenase D (Sigma, 1188866001) with 10% v/v of papain (Roche, 10108014001) in HBSS (Gibco, 14170–112) for 90 min, with vortexing every 30 min. Cells were centrifuged at 400 × g, and the pellet was resuspended in Enzyme T (Sigma, 10109886001) (soybean trypsin inhibitor made in 1:1 bovine serum albumin and DMEM/F12 media (Thermo-Fisher, 10565161) supplemented with 10% fetal bovine serum (FBS) (Hyclone, SH30088.03) and 1% pen/ strep (Sigma, P4333)) to stop the enzymatic reaction. Digested tissues were triturated using a 1 mL pipette tip and passed through a 70-μm nylon mesh cell strainer (Sigma, CLS431751-50EA), with a subsequent wash using flow buffer (0.5% bovine serum albumin with 0.02% glucose (Sigma, G7528) made in 1 × DPBS). The resultant suspension was further processed for flow cytometry (see details below).

### Flow cytometry

Freshly dissociated paw skin cells from plantar skin were suspended in ice-cold sterile 1 × DPBS and centrifuged at 400 × g for 3 min. The cells were resuspended in flow buffer (0.5% bovine serum albumin with 0.02% glucose (Sigma, G7528) made in 1 × DPBS), with blocking antibody (anti-CD16/32 purified (1:2000) (eBioscience, 16016185)) for 20 min to block Fc receptors. Samples were then incubated with pre-conjugated extracellular flow antibodies, F4/80 (BioLegend 123141) and CD3 (eBioscience, 56003282) for 45 min. Samples were washed with flow buffer, centrifuged at 400 × g for 3 min, and resuspended in a DAPI wash for 5 min. Samples were centrifuged at 400 × g for 3 min, washed twice using flow buffer, and resuspended in flow buffer. Appropriate compensation controls and isotypes were used for determination and gating. After gating DAPI-positive cells (to determine debris), α7^tdT^ (tdTomato^+^ cre driven nAChRs) cells were gated to determine the overlap with F4/80 and CD3. Stained samples were analyzed using a Special Order (4-laser) Becton–Dickinson Fortessa analyzer (Red Oaks, CA), and data were analyzed using FlowJo and FCS Express software (De NoVo Software, Los Angeles, CA). Experimenters were blinded to sex and treatments.

### Data analysis and statistics

Data are expressed as mean ± standard error of the mean (S.E.M.). Statistical analyses were performed using Prism (v. 9, GraphPad Software Inc., La Jolla, CA, U.S.A.). Normality was assessed in all data sets using the Kolmogorov–Smirnov test. For normally distributed data, paired t-test was used to compare the means of two dependent groups with one variable, two-way analysis of variance (ANOVA) test was used to compare the means of two or more independent groups with two independent variables, and repeated measures two-way ANOVA was used for complete data sets to compare two or more dependent groups with two independent variables. Šidák’s, Tukey’s, or Dunnett’s posthoc multiple comparison tests were used for independent comparisons of selected data, for comparing between groups, and to compare experimental groups to a single control group, respectively. For non-normally distributed data with four independent groups and one variable, we used the Kruskal–Wallis test followed by Dunn’s posthoc multiple comparison test for independent comparison of selected data. For categorical type of data, we employed non-parametric Wilcoxon matched-pairs signed rank test to compare two dependent groups with one variable, non-parametric Mann–Whitney multiple comparisons test, corrected with the Holm-Šídák method, to compare two independent groups with two independent variables, and the non-parametric Kruskal–Wallis test followed by Dunn’s posthoc multiple comparisons test to compare four dependent groups with one variable. *P*-values < 0.05 were recognized as significant. See Table 1 for a detailed description of statistics.

**Table 1:** Statistical analyses

Figure	Type of Data	Type of Test	Sample Size	Statistical Data
Figure 1				
1c (Acetone; injected vs non-injected paw)	Categorical	Non-parametric Wilcoxon matched-pairs signed rank test	vehicle predrug = 9 mice	Wilcoxon matched-pairs signed rank test: DMSO non-injected paw vs. DMSO injected paw pre-drug, *p* = 0039
1c (Acetone; injected paw max. effect vs uninjected paw)	Categorical	Non-parametric Mann–Whitney multiple comparisons test, Holm-Šídák method	vehicle uninjected paw day 7 = 9 mice; 4R 15 mg/kg day 7 = 7 mice	Mann–Whitney test, DMSO non-injected paw day 7 vs. 4R 15 mg/kg day 7 injected paw, adjusted *p* = 0.0086
1d (Hargreaves; injected vs non-injected paw)	Normal	Two-way ANOVA followed by Tukey's multiple comparisons test	vehicle (predrug, 2–5 h., day 2) = 6 mice; vehicle day 8 = 9 mice; 4R 1 mg/kg (all time points) = 6 mice; 4R 6 mg/kg (predrug, 2–5 h., day 2) = 6 mice; 4R 6 mg/kg day 8 = 8 mice; 4R 15 mg/kg (predrug, 2–5 h., day 2) = 6 mice; 4R 15 mg/kg day 8 = 7 mice	Two-way ANOVA
				time: F (3, 172) = 37.71; *p* < 0.0001
				treatment: F (7, 172) = 173.3; *p* < 0.0001
				interaction: F (21, 172) = 6.204; *p* < 0.0001
				*posthoc*: Tukey’s multiple comparisons test
				CFA-injected paw vs uninjected paw in veh conditions: *p* < 0.0001 (predrug); *p* < 0.0001 (2–5 h, 6 mg/kg and 15 mg/kg); *p* < 0.0001 (day 2, 6 mg/kg); *p* < 0.0001 (day 8, 1 mg/kg); *p* = 0.2131 (day 8, 6 mg/kg); *p* = 0.2354 (day 8, 15 mg/kg)
1d (Hargreaves; injected paw time course)	Normal	Two-way ANOVA followed by Dunnett's multiple comparisons test	vehicle (predrug, 2–5 h., day 2) = 6 mice; vehicle day 8 = 9 mice; 4R 1 mg/kg (all time points) = 6 mice; 4R 6 mg/kg (predrug, 2–5 h., day 2) = 6 mice; 4R 6 mg/kg day 8 = 8 mice; 4R 15 mg/kg (predrug, 2–5 h., day 2) = 6 mice; 4R 15 mg/kg day 8 = 7 mice	Two-way ANOVA
				time: F (3, 86) = 59.20; *p* < 0.0001
				treatment: F (3, 86) = 17.87; *p* < 0.0001
				interaction: F (9, 86) = 3.671; *p* = 0.0006
				*posthoc*: Dunnett’s multiple comparisons test
				2–5 h: *p* = 0.0395 for DMSO vs. 4R 6 mg/kg; *p* < 0.0001 for DMSO vs. 4R 15 mg/kg
				day 2: *p* = 0.0035 for DMSO vs. 4R 6 mg/kg
				day 8: *p* = 0.0067 for DMSO vs. 4R 1 mg/kg; *p* < 0.0001 for DMSO vs. 4R 6 mg/kg and DMSO vs. 4R 15 mg/kg
1d (Hargreaves; non-injected paw time course)	Normal	Two-way ANOVA	vehicle (predrug, 2–5 h., day 2) = 6 mice; vehicle day 8 = 9 mice; 4R 1 mg/kg (all time points) = 6 mice; 4R 6 mg/kg (predrug, 2–5 h., day 2) = 6 mice; 4R 6 mg/kg day 8 = 8 mice; 4R 15 mg/kg (predrug, 2–5 h., day 2) = 6 mice; 4R 15 mg/kg day 8 = 7 mice	Two-way ANOVA
				time: F (3, 86) = 2.964; *p* = 0.0366
				treatment: F (3, 86) = 1.450; *p* = 0.2338
				interaction: F (9, 86) = 0.4422; *p* = 0.9083
1e (von Frey; injected vs non-injected paw)	Normal	Two-way ANOVA followed by Tukey's multiple comparisons test	vehicle (predrug, 2–5 h.) = 6 mice; vehicle day 2 = 4 mice; vehicle day 8 = 7 mice; 4R 1 mg/kg (predrug, 2–5 h) = 6 mice; 4R 1 mg/kg (day 2, day 8) = 5 mice; 4R 6 mg/kg (predrug, 2–5 h., day 8) = 6 mice; 4R 6 mg/kg day 2 = 5 mice; 4R 15 mg/kg (predrug, 2–5 h., day 8) = 6 mice; 4R 15 mg/kg day 2 = 4 mice	Two-way ANOVA
				time: F (3, 148) = 2.378; *p* = 0.0722
				drug treatments: F (7, 148) = 121.8; *p* < 0.0001
				interaction: F (21, 148) = 0.7112; *p* = 0.8167
				*posthoc*: Tukey’s multiple comparisons test
				CFA-injected paw vs uninjected paw in veh conditions; *p* < 0.0001 (predrug); *p* < 0.0001 (2–5 h, 1 mg/kg and 15 mg/kg); *p* < 0.0001 (day 2, 1 mg/kg, 6 mg/kg)
1e (von Frey; injected paw time course)	Normal	Two-way ANOVA followed by Dunnett's multiple comparisons test	vehicle (predrug, 2–5 h.) = 6 mice; vehicle day 2 = 4 mice; vehicle day 8 = 7 mice; 4R 1 mg/kg (predrug, 2–5 h) = 6 mice; 4R 1 mg/kg (day 2, day 8) = 5 mice; 4R 6 mg/kg (predrug, 2–5 h., day 8) = 6 mice; 4R 6 mg/kg day 2 = 5 mice; 4R 15 mg/kg (predrug, 2–5 h., day 8) = 6 mice; 4R 15 mg/kg day 2 = 4 mice	Two-way ANOVA
				time: F (3, 74) = 4.861; *p* = 0.0038
				drug treatments: F (3, 74) = 4.891; *p* = 0.0037
				interaction: F (9, 74) = 1.062; *p* = 0.4008
				*posthoc*: Dunnett’s multiple comparisons test
				2–5 h: *p* = 0.0403 for DMSO vs. 4R 1 mg/kg; *p* = 0.0476 for DMSO vs. 4R 15 mg/kg
				day 2: *p* = 0.0487 for DMSO vs. 4R 1 mg/kg; *p* = 0.0479 for DMSO vs. 4R 6 mg/kg day 8: *p* = 0.0067 for DMSO vs. 4R 1 mg/kg; *p* < 0.0001 for DMSO vs. 4R 6 mg/kg and DMSO vs. 4R 15 mg/kg
1e (von Frey; non-injected paw time course)	Normal	Two-way ANOVA	vehicle (predrug, 2–5 h.) = 6 mice; vehicle day 2 = 4 mice; vehicle day 8 = 7 mice; 4R 1 mg/kg (predrug, 2–5 h) = 6 mice; 4R 1 mg/kg (day 2, day 8) = 5 mice; 4R 6 mg/kg (predrug, 2–5 h., day 8) = 6 mice; 4R 6 mg/kg day 2 = 5 mice; 4R 15 mg/kg (predrug, 2–5 h., day 8) = 6 mice; 4R 15 mg/kg day 2 = 4 mice	Two-way ANOVA
				time: F (3, 74) = 0.7107; *p* = 0.5487
				treatment: F (3, 74) = 0.5522; *p* = 0.6483
				interaction: F (9, 74) = 0.7197; *p* = 0.6892
Figure 2				
2b (Acetone; injected vs. non-injected paw; day 2)	Categorical	Non-parametric Mann–Whitney multiple comparisons test, Holm-Šídák method	vehicle and 4R 15 mg/kg = 6 mice	Mann–Whitney multiple comparisons test, Holm-Šídák method; DMSO injected paw vs. DMSO uninjected paw: adjusted *p* = 0.0043; DMSO uninjected vs 4R 15 mg/kg in injected paw: adjusted *p* = 0.0152
2b (Acetone; veh vs. 4R; day 2)	Categorical	Non-parametric Mann–Whitney multiple comparisons test, Holm-Šídák method	vehicle and 4R 15 mg/kg = 6 mice	Mann–Whitney multiple comparisons test, Holm-Šídák method; DMSO vs. 4R 15 mg/kg in injected paw: adjusted *p* = 0.0258
2c (Hargreaves; injected vs non-injected paw; day 2)	Normal	Two-way ANOVA followed by Šídák's multiple comparisons test	vehicle and 4R 15 mg/kg = 6 mice	Two-way ANOVA
				paw side: F (1, 20) = 384.3; *p* < 0.0001
				treatment: F (1, 20) = 38.57; *p* < 0.0001
				interaction:F (1, 20) = 26.88; *p* < 0.0001
				*posthoc*: Šídák’s multiple comparisons test
				adjusted *p* < 0.0001 for CFA-injected paw vs uninjected paw in veh conditions;
2c (Hargreaves; 4R injected vs. DMSO non-injected paw; day 2)	Normal	Two-way ANOVA followed by Tukey's multiple comparisons test	vehicle and 4R 15 mg/kg = 6 mice	Two-way ANOVA
				paw side: F (1, 20) = 384.3; *p* < 0.0001
				treatment: F (1, 20) = 38.57; *p* < 0.0001
				interaction:F (1, 20) = 26.88; *p* < 0.0001
				*posthoc*: Tukeys multiple comparisons test
				adjusted *p* < 0.0001 for 4R 15 mg/kg CFA-injected paw vs uninjected paw in veh conditions
2c (Hargreaves;veh vs 4R; day 2)	Normal	Two-way ANOVA followed by Šídák's multiple comparisons test	vehicle and 4R 15 mg/kg = 6 mice	Two-way ANOVA
				paw side: F (1, 20) = 384.3; *p* < 0.0001
				treatment: F (1, 20) = 38.57; *p* < 0.0001
				interaction: F (1, 20) = 26.88; *p* < 0.0001
				*posthoc*: Šídák’s multiple comparisons test
				*p* < 0.0001 for DMSO vs. 4R 15 mg/kg in injected paw
				*p* = 0.7260 for DMSO vs. 4R 15 mg/kg in non-injected paw
2d (von Frey; injected vs non-injected paw; day 2)	Normal	Two-way ANOVA followed by Šídák's multiple comparisons test	vehicle and 4R 15 mg/kg = 6 mice	Two-way ANOVA
				paw side: F (1, 20) = 277.2, *p* < 0.0001
				treatment: F (1, 20) = 1.213; *p* = 0.2838
				interaction:F (1, 20) = 1.213; *p* = 0.2838
				*posthoc*: Šídák’s multiple comparisons test
				*p* < 0.0001 for CFA-injected paw vs uninjected paw in veh conditions
2d (von Frey; veh vs 4R; day 2)	Normal	Two-way ANOVA followed by Šídák's multiple comparisons test	vehicle and 4R 15 mg/kg = 6 mice	Two-way ANOVA
				paw side:F (1, 20) = 277.2; *p* < 0.0001
				treatment: F (1, 20) = 1.213; *p* = 0.2838
				interaction: F (1, 20) = 1.213; *p* = 0.2838
				*posthoc*: Šídák’s multiple comparisons test
				*p* = 0.2518 for DMSO vs. 4R 15 mg/kg in injected paw
				*p* > 0.9999 for DMSO vs. 4R 15 mg/kg in non-injected paw
2e (Acetone; injected vs non-injected paw; day 7)	Categorical	Non-parametric Mann–Whitney multiple comparisons test, Holm-Šídák method	vehicle and 4R 15 mg/kg = 6 mice	Mann–Whitney multiple comparisons test, Holm-Šídák method; DMSO injected paw vs. DMSO uninjected paw: adjusted *p* = 0.0043, DMSO uninjected vs 4R 15 mg/kg in injected paw: adjusted *p* = 0.0043
2f (Hargreaves; injected vs non-injected paw; day 7)	Normal	Two-way ANOVA followed by Šídák's multiple comparisons test	vehicle and 4R 15 mg/kg = 6 mice	Two-way ANOVA
				paw side: F (1, 20) = 42.74; *p* < 0.0001
				treatment: F (1, 20) = 7.807; *p* = 0.0112
				interaction:F (1, 20) = 5.150; *p* = 0.0345
				*posthoc*: Šídák’s multiple comparisons test
				adjusted *p* < 0.0001 for CFA-injected paw vs uninjected paw in veh conditions,
2f (Hargreaves; 4R injected vs. DMSO non-injected paw; day 7)	Normal	Two-way ANOVA followed by Tukey's multiple comparisons test	vehicle and 4R 15 mg/kg = 6 mice	Two-way ANOVA
				paw side: F (1, 20) = 42.72; *p* < 0.0001
				treatment: F (1, 20) = 7.807; *p* = 0.0112
				interaction:F (1, 20) = 5.150; *p* = 0.0345
				*posthoc*: Tukeys multiple comparisons test
				adjusted *p* = 0.0679 for 4R 15 mg/kg CFA-injected paw vs uninjected paw in veh conditions
2f (Hargreaves;veh vs 4R; day 7)	Normal	Two-way ANOVA followed by Šídák's multiple comparisons test	vehicle and 4R 15 mg/kg = 6 mice	Two-way ANOVA
				paw side: F (1, 20) = 42.74; *p* < 0.0001
				treatment: F (1, 20) = 7.807; *p* = 0.0112
				interaction: F (1, 20) = 5.150; *p* = 0.0345
				*posthoc*: Šídák’s multiple comparisons test
				*p* = 0.0037 for DMSO vs. 4R 15 mg/kg in injected paw
				*p* = 0.9185 for DMSO vs. 4R 15 mg/kg in non-injected paw
2 g (von Frey; injected vs non-injected paw; day 7)	Normal	Two-way ANOVA followed by Šídák's multiple comparisons test	vehicle and 4R 15 mg/kg = 6 mice	Two-way ANOVA
				paw side: F (1, 20) = 74.43; *p* < 0.0001
				treatment: F (1, 20) = 0.8013; *p* = 0.3814
				interaction: F (1, 20) = 0.01463; *p* = 0.9049
				*posthoc*: Šídák’s multiple comparisons test
				*p* < 0.0001 for CFA-injected paw vs uninjected paw in veh conditions
2g (von Frey; veh vs 4R; day 7)	Normal	Two-way ANOVA followed by Šídák's multiple comparisons test	vehicle and 4R 15 mg/kg = 6 mice	Two-way ANOVA
				paw side: F (1, 20) = 74.43; *p* < 0.0001
				treatment: F (1, 20) = 0.8013; *p* = 0.3814
				interaction: F (1, 20) = 0.01463; *p* = 0.9049
				*posthoc*: Šídák’s multiple comparisons test
				*p* = 0.7304 for DMSO vs. 4R 15 mg/kg in injected paw
				*p* = 0.8320 for DMSO vs. 4R 15 mg/kg in non-injected paw
Figure 3				
3b (thickness of injected vs non-injected paw, male)	Normal	Two-way ANOVA followed by Tukey's multiple comparisons test	vehicle (predrug, 2–5 h., day 2) = 16 mice; vehicle (day 3, day 7) = 12 mice; vehicle day 8 = 9; 4R 1 mg/kg (predrug, 2–5 h., day 2) = 14 mice; 4R 1 mg/kg (day 3, day 7) = 12 mice; 4R 1 mg/kg day 8 = 6 mice; 4R 6 mg/kg (predrug, 2–5 h., day 2) = 16 mice; 4R 6 mg/kg (day 3, day 7) = 12 mice; 4R 6 mg/kg day 8 = 8 mice; 4R 15 mg/kg (predrug, 2–5 h., day 2) = 14 mice; 4R 15 mg/kg (day 3, day 7) = 12 mice; 4R 15 mg/kg day 8 = 7 mice	Two-way ANOVA
				time: F (5, 564) = 45.20; *p* < 0.0001
				drug treatments: F (7, 564) = 447.0; *p* < 0.0001
				interaction: F (35, 564) = 8.479; *p* < 0.0001
				*posthoc*: Tukey’s multiple comparisons test
				CFA-injected paw vs uninjected paw in veh conditions; *p* < 0.0001 (predrug); *p* < 0.0001 (day 7, 4R 6 mg/kg)
3b (injected paw thickness time course, male)	Normal	Two-way ANOVA followed by Dunnett's multiple comparisons test	vehicle (predrug, 2–5 h., day 2) = 16 mice; vehicle (day 3, day 7) = 12 mice; vehicle day 8 = 9; 4R 1 mg/kg (predrug, 2–5 h., day 2) = 14 mice; 4R 1 mg/kg (day 3, day 7) = 12 mice; 4R 1 mg/kg day 8 = 6 mice; 4R 6 mg/kg (predrug, 2–5 h., day 2) = 16 mice; 4R 6 mg/kg (day 3, day 7) = 12 mice; 4R 6 mg/kg day 8 = 8 mice; 4R 15 mg/kg (predrug, 2–5 h., day 2) = 14 mice; 4R 15 mg/kg (day 3, day 7) = 12 mice; 4R 15 mg/kg day 8 = 7 mice	Two-way ANOVA
				time: F (5, 282) = 50.75; *p* < 0.0001
				drug treatments: F (3, 282) = 12.64; *p* < 0.0001
				interaction:F (15, 282) = 1.301; *p* = 0.2007
				*posthoc*: Dunnett’s multiple comparisons test
				day 2: *p* = 0.0212 for DMSO vs. 4R 15 mg/kg
				day 3: *p* = 0.0061 for DMSO vs. 4R 6 mg/kg; *p* = 0.0165 for DMSO vs. 4R 15 mg/kg
				day 7: *p* = 0.0006 for DMSO vs. 4R 6 mg/kg; *p* = 0.0061 for DMSO vs. 4R 15 mg/kg
				day 8: *p* = 0.0172 for DMSO vs. 4R 6 mg/kg; *p* = 0.0152 for DMSO vs. 4R 15 mg/kg
3c (thickness of injected vs non-injected paw, female)	Normal	Two-way RM ANOVA followed by Tukey’s multiple comparisons test	vehicle and 4R 15 mg/kg = 6 mice	Two-way RM ANOVA
				time: F (1.448, 28.95) = 88.41; *p* < 0.0001
				drug treatments: F (3, 20) = 520.4; *p* < 0.0001
				time X drug treatments: F (15, 100) = 33.57; *p* < 0.0001
				*posthoc*: Tukey's multiple comparisons test
				pre-drug: *p* < 0.0001 for CFA-injected paw vs uninjected paw in veh conditions
				time: F (5, 60) = 70.27; *p* < 0.0001
				drug treatments: F (1, 60) = 0.5000; *p* = 0.4822
				interaction: F (5, 60) = 2.675; *p* = 0.0301
3f (injected paw glabrous skin thickness)	Normal	Two-way ANOVA followed by Šídák's multiple comparisons test	Vehicle—male = 6 mice	Two-way ANOVA
			Vehicle—female = 6 mice	drug treatment: F (1, 19) = 4.647; *p* = 0.0441
			4R—male = 5 mice	sex: F (1, 19) = 0.294; *p* = 0.5936
			4R—female = 6 mice	interaction: F (1, 19) = 0.056; *p* = 0.8140
				*posthoc*: Šídák’s multiple comparisons test
				*p* = 0.3617 for Males Vehicle vs. Males 4R
				*p* = 0.1881 for Females Vehicle vs. Females 4R
3g (injected paw subdermal cell count)	Normal	Two-way ANOVA followed by Šídák's multiple comparisons test	Vehicle—male = 6 mice	Two-way ANOVA
			Vehicle—female = 6 mice	drug treatment: F (1, 19) = 7.495; *p* = 0.0131
			4R—male = 5 mice	sex: F (1, 19) = 0.4968; *p* = 0.48985
			4R—female = 6 mice	interaction: F (1, 19) = 7.073; *p* = 0.0155
				*posthoc:* Šídák’s multiple comparisons test
				*p* = 0.0028 for Males Vehicle vs. Male 4R
				*p* = 0.9980 for Females Vehicle vs. Females 4R
				*p* = 0.0489 for Males Vehicle vs. Females Vehicle
				*p* = 0.3484 for Males 4R vs. Females 4R
Figure 4				
4b (Acetone)	Categorical	Non-parametric Kruskal–Wallis test followed by Dunn’s multiple comparisons test	MLA + DMSO, MLA + 4R, Saline + 4R and Saline + DMSO = 6 mice	Kruskal–Wallis test: *p* = 0.0076; posthoc: Dunn’s mutiple comparisons test: Saline + 4R vs. MLA + 4R *p* = 0.0041; Saline + DMSO vs. MLA + DMSO *p* = 0.7503;
4c (Hargreaves)	Non-normal	Non-parametric Kruskal–Wallis test followed by Dunn's multiple comparisons test	MLA + DMSO, MLA + 4R, Saline + 4R and Saline + DMSO = 6 mice	Kruskal–Wallis test: *p* = 0.0005; posthoc: Dunn's mutiple comparisons test: Saline + 4R vs. MLA + 4R *p* = 0.0058; Saline + DMSO vs. MLA + DMSO *p* = 0.0825;
4d (thickness of injected vs non-injected paw, male)	Normal	Two-way RM ANOVA followed by Tukey's multiple comparisons test	MLA + DMSO = 6 mice, MLA + 4R = 6 mice, Saline + 4R = 6 mice and Saline + DMSO = 3–6 mice	Two-way ANOVA
				time: F (1.561, 57.76) = 37.40; *p* < 0.0001
				drug treatments: F (7, 37) = 290.5; *p* < 0.0001
				interaction: F (35, 185) = 7.757; *p* < 0.0001
				*posthoc*: Tukey’s multiple comparisons test
				pre-drug: *p* < 0.0001 for CFA-injected paw vs uninjected paw in veh conditions
4d (injected paw thickness time course, male)	Normal	Two-way RM ANOVA followed by Tukey’s multiple comparisons test	MLA + DMSO = 6 mice, MLA + 4R = 6 mice, Saline + 4R = 6 mice and Saline + DMSO = 3–6 mice	Two-way ANOVA
				time: F (1.403, 28.06) = 52.55; *p* < 0.0001
				drug treatments: F (3, 20) = 2.550; *p* = 0.0846
				interaction: F (15, 100) = 1.651; *p* = 0.0737
				*posthoc*: Tukey’s multiple comparisons test
				day 2: *p* = 0.0076 for Saline + 4R vs. Saline + DMSO
				day 3: *p* = 0.0026 for Saline + 4R vs. Saline + DMSO
				day 7: *p* = 0.0200 for Saline + 4R vs. Saline + DMSO; *p* = 0.0069 for MLA + 4R vs. Saline + 4R
				day 8: *p* = 0.0116 for Saline + 4R vs. Saline + DMSO; *p* = 0.0043 for MLA + 4R vs. Saline + 4R
Figure 5				
5c (injected paw subdermal α7^tdT^ cell count)	Normal	Two-way ANOVA	Vehicle—male = 5 mice	Two-way ANOVA
			Vehicle—female = 4 mice	drug treatments: F(1, 13) = 0.001727; *p* = 0.9675
			4R—male = 4 mice	
			4R—female = 4 mice	sex: F(1, 13) = 0.008159; *p* = 0.9294
				interaction: F(1, 13) = 0.6627; *p* = 0.4303
5d (injected paw subdermal Iba-1^+^ cell count	Normal	Two-way ANOVA	Vehicle—male = 4 mice	Two-way ANOVA
			Vehicle—female = 4 mice	drug treatments: F(1, 12) = 0.2515; *p* = 0.6251
			4R—male = 4 mice	sex: F(1, 12) = 0.02600; *p* = 0.6251
			4R—female = 4 mice	interaction: F(1, 12) = 0.8920; *p* = 0.3636
5e (injected paw subdermal colocalized cell count	Normal	Two-way ANOVA	Vehicle—male = 5 mice	Two-way ANOVA
			Vehicle—female = 4 mice	drug treatments: F(1, 13) = 0.6376; *p* = 0.4389
			4R—male = 4 mice	sex: F(1, 12) = 0.05774; *p* = 0.8138
			4R—female = 4 mice	interaction: F(1, 12) = 0.5835; *p* = 0.4586
5h (injected paw subdermal α7^tdT^ cell count	Normal	Paired t-test	CFA-injected—male = 4 mice	Paired t-test (F4/80)
			uninjected—male = 4 mice	*p* value: 0.5122
			CFA-injected—female = 4 mice	t, df: t = 0.6904, df = 7
			uninjected—female = 4 mice	Paired t-test (CD3 +)
				*p* value: 0.9088
				t, df: t = 0.1188, df = 7

Detailed information about data structure, statistical tests, sample sizes and statistical results

*F*_*(DFn, DFd)*_ Degree of freedom for the numerator of the F ratio, for the denominator of the F ratio, *df* Degrees of freedom

## Results

### Systemic administration of 4R tobacco cembranoid reduces inflammation-induced peripheral hypersensitivity in male mice

The CFA mouse model of persistent inflammatory pain was used to test the hypothesis that systemic administration of 4R tobacco cembranoid decreases inflammation-induced peripheral hypersensitivity. CFA injection in the paw has been shown to cause swelling and hypersensitivity to cold, heat, and tactile stimuli [53, 54]. The potential antinociceptive effects of systemic administration of 4R on cold, heat, and tactile sensitivity in the CFA-injected and uninjected hind paws were measured at different time points (pre-drug, and 2–5 h, day 2, day 8 after 4R or vehicle administration) using acetone, Hargreaves, and von Frey test, respectively (Fig. 1a-e). Three doses of 4R (1, 6, and 15 mg/kg by body weight) or vehicle, injected subcutaneously one day after the CFA injection, were evaluated (Fig. 1b).

Consistent with previous reports [53, 55], subcutaneous injection of CFA into the hind paw induced hypersensitivity to cold, heat, and tactile stimuli in the injected paw, compared to the uninjected hind paw (Fig. 1c-e). Thus, prior to 4R treatment (pre-drug condition), the response score to acetone paw stimulation was significantly (*p* < 0.001) higher (Fig. 1c), and the withdrawal latencies to heat stimulation were significantly (*p* < 0.0001) shorter (Fig. 1d) in CFA-treated hind paws, compared to the untreated hind paws. Similarly, paw withdrawal thresholds in response to tactile stimulation were significantly (*p* < 0.0001) lower in the CFA-injected paw compared to the uninjected hind paw (Fig. 1e). As expected, CFA-induced persistent hypersensitivity to cold, heat, and tactile stimulation was observed in the injected hind paws of control mice (systemically treated with vehicle) for 8 days after CFA injection.

As illustrated in Fig. 1c-e, a single injection of 4R reduces CFA-induced hypersensitivity in a dose and time-dependent manner. Following the administration of the lowest dose of 4R (1 mg/kg), for example, a significant (*p* < 0.01) decrease in response scores to acetone stimulation was observed in the CFA-injected paw 2–5 h after 4R treatment compared to vehicle-treated mice (Fig. 1c). However, the effect of a single injection of 4R (1 mg/kg) in cold hypersensitivity,

was transient as responses in 4R-treated animals were indistinguishable from control vehicle-treated mice when tested on days 2 and 7 after 4R treatment. In contrast to the transient effects observed after administration of the lowest dose of 4R, the highest dose of 4R tested (15 mg/kg) resulted in long-lasting decreases in CFA-induced cold hypersensitivity, manifested as significantly (*p* < 0.0001) lower response scores to acetone stimulation of the injected paw, compared to response scores in vehicle-treated mice, in all time-points, tested (Fig. 1c). Administration of 4R at 6 mg/kg of weight resulted in significant (*p* < 0.001) decreases in response scores to acetone stimulation in the CFA-treated hind paw 2–5 h and on day 7 after the single injection of 4R but not on day 2 after treatment. Further analysis revealed that responses to cold stimulation of the injected paw at maximum analgesia (observed 7 days after a single dose of 4R 15 mg/kg) are significantly (*p* < 0.01) higher than responses in the uninjected control paw (Fig. 1c), demonstrating that 4R treatment partially reverses CFA-induced cold allodynia.

Evaluation of CFA-induced hypersensitivity to heat stimuli revealed that 4R also decreases heat hypersensitivity in the inflamed paw in a time and dose-dependent manner (Fig. 1d). Compared to vehicle-treated animals, mice treated with the lowest 4R dose (1 mg/kg), displayed a significant (*p* < 0.01) increase in paw withdrawal latency in response to heat stimulation (less hypersensitivity) of the CFA-injected paw on day 8 (Fig. 1d). At this dose, no effect on CFA-induced thermal hypersensitivity was observed 2–5 h or day 2 after 4R treatment (Fig. 1d). In contrast, administration of the highest dose of 4R (15 mg/kg) showed strong anti-hyperalgesic effects in response to heat stimulation of the injected paw 2–5 h and day 8 (*p* < 0.0001), but not on day 2 after 4R treatment. Furthermore, mice treated with 4R at 6 mg/kg of body weight displayed time-dependent anti-hyperalgesic effects with increasing paw withdrawal latency (less hypersensitivity) compared to vehicle-treated animals as time after treatment progressed. Additional analysis showed no significant differences (*p* > 0.05) in responses to heat stimulation between the uninjected control paw and 4R 6 mg/kg or 15 mg/kg treated CFA-injected paw on day 8 (Fig. 1d). In contrast, paw withdrawal latencies were significantly (*p* < 0.0001) lower 2–5 h after 4R treatment (6 mg/kg and 15 mg/kg) and on day 2 after 4R (6 mg/kg), when compared to the uninjected control paw (Fig. 1d). These results demonstrate that CFA-induced heat hypersensitivity is reversed 8 days after a single dose of 4R treatment (6 mg/kg and 15 mg/kg), but it is only partially reversed by the 1 mg/kg dose at this time-point or after any dose (6 mg/kg and 15 mg/kg) at earlier time-points (2–5 h and day 2).

Lastly, analysis of inflammation-induced hypersensitivity to tactile stimulation revealed a small but statistically significant (*p* < 0.05) increase (less hypersensitivity) in paw withdrawal thresholds in the CFA-injected paw 2–5 h after 4R (1 mg/kg and 15 mg/kg) treatment and 2 days after 4R (1 mg/kg and 6 mg/kg) administration when compared to vehicle-treated mice (Fig. 1e). When compared to the uninjected control paw, paw withdrawal thresholds were significantly (*p* < 0.0001) lower (more hypersensitive) at all time-points and 4R doses evaluated (Fig. 1e). These results indicate that 4R-treated mice are still clearly allodynic, even though there is a very small improvement in their von Frey thresholds.

Notably, measurement of responses to cold, heat, and tactile stimulation of the uninjured hind paw showed that responses in all modalities are indistinguishable in vehicle- and 4R-treated mice throughout the duration of the experiment and independent of the dose tested. These results are significant as they indicate that baseline nociception (in the absence of inflammation) and locomotor responses to peripheral stimuli are unaffected by 4R systemic treatment (Fig. 1c-e). Collectively, these results demonstrate that a single systemic administration of 4R decreases inflammation-induced peripheral hypersensitivity without affecting baseline responses in the uninjured paw.

### Systemic 4R administration reduces inflammation-induced thermal but not tactile hypersensitivity in female mice

**Fig. 2:**
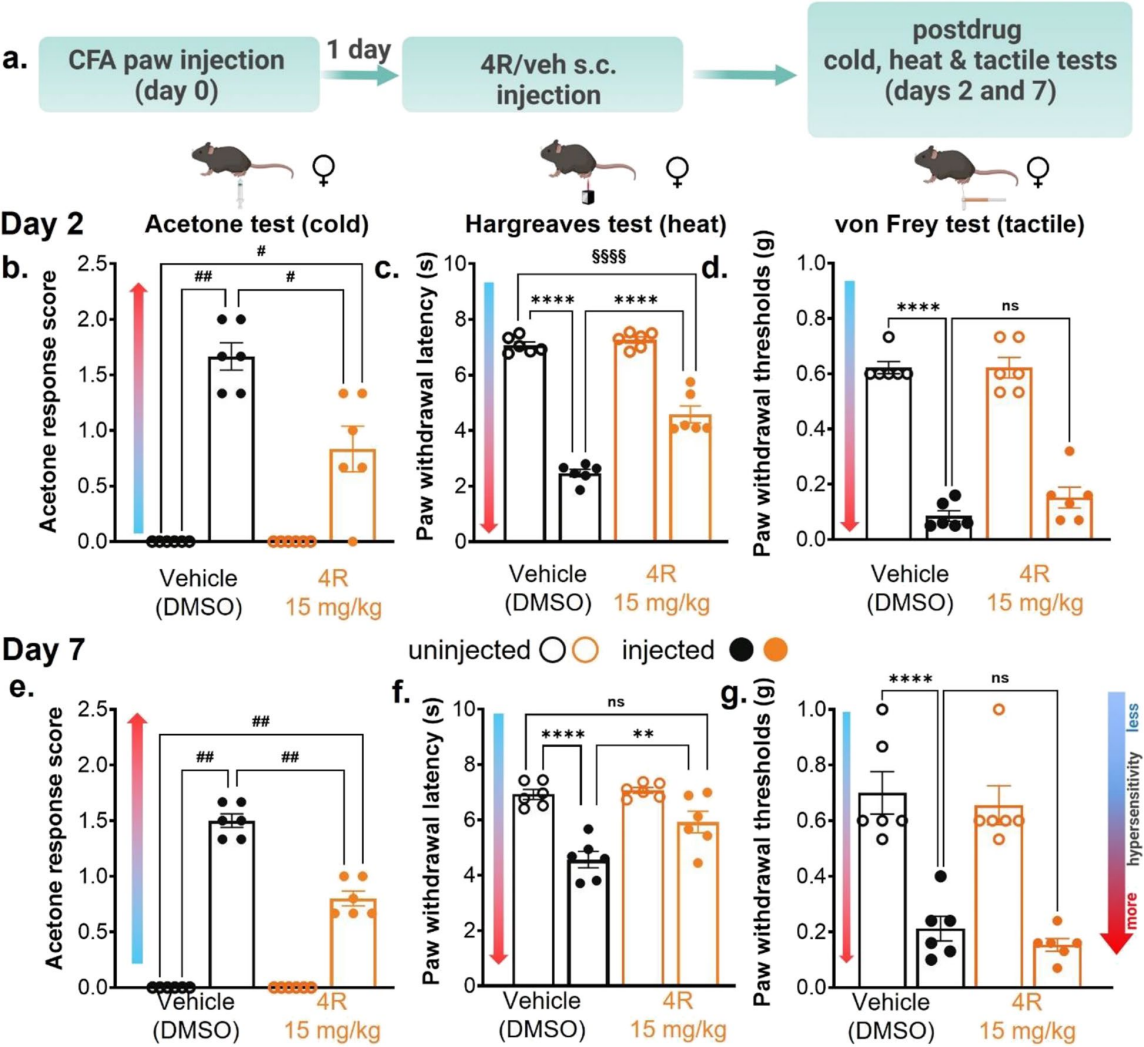
Systemic 4R administration reduces inflammation-induced thermal but not tactile hypersensitivity in female mice (**a**) Experimental timeline for acetone (cold), Hargreaves (heat) and von Frey (tactile) tests in CFA-injected and uninjected hind paws at days 2 and 7 after 4R (15 mg/kg) or vehicle administration in females. Acetone response score (**b**, **e**), withdrawal latency to heat stimulation (**c**, **f**) and withdrawal threshold to tactile stimulation (**d**, **g**) on experimental days 2 and 7, respectively. All values are expressed as mean ± SEM. *n* = 6 animals per treatment and test. Two-way ANOVA followed by Šídák’s multiple comparison test; *p* < 0.01 (**); *p* < 0.001 (***); *p* < 0.0001 (****) or Tukey’s multiple comparisons test; adjusted *p* < 0.0001 (§§§§). Mann–Whitney multiple comparisons test, Holm-Šídák method; adjusted *p* < 0.05 (#); adjusted *p* < 0.01 (##). Not significant = ns

Previous studies report sex-related mechanistic differences in rodent inflammatory processes that influence nociception and hyperalgesia [56–60]. These findings stress the importance of studying male and female subjects in preclinical pain studies. In line with this, the next set of experiments aimed to determine whether the 4R anti-hyperalgesic effects described above in males are also observed in females using the same experimental approach (Fig. 2a). As illustrated in Fig. 2b-g, evaluation of the responses to acetone, Hargreaves, and von Frey tests showed that female control mice display cold, heat, and tactile hypersensitivity in CFA-injected paws compared to their respective uninjected paws on days 2 and 7 after systemic vehicle treatment. Thus, response scores to acetone stimulation were significantly (*p* < 0.01) higher in CFA-injected hind paws than in the uninjected hind paws (Fig. 2b, e). Similarly, paw withdrawal latencies and thresholds to heat and tactile stimulation of the CFA-injected paw were significantly (*p* < 0.0001) shorter and lower, respectively, than those measured in the uninjected hind paws (Fig. 2c, d, f, and g).

To investigate the potential effects of 4R in females, we used the 15 mg/kg dose, which consistently reduced inflammation-induced hyperalgesia in males (Fig. 1). These behavioral experiments revealed that a single s.c. injection of 4R (15 mg/kg) in female mice significantly (*p* < 0.01) decreases CFA-induced cold and heat hypersensitivity compared to animals treated with the vehicle on both days 2 and 7 after systemic treatment (Fig. 2b-c, e–f). Thus, on both days tested, response scores to acetone were significantly (*p* < 0.05) lower in the CFA-injected paw of 4R-treated mice than in the injected paws of the vehicle-treated group (Fig. 2b, e). Similarly, paw withdrawal latencies to heat stimulation of the injected paw on both days were significantly (*p* < 0.01) longer in 4R-treated mice than in those treated with vehicle (Fig. 2c, f). Similar to what we observed in males (Fig. 1c-d), CFA-induced heat hypersensitivity was reversed 7 days after a single dose of 4R treatment (15 mg/kg). Thus, there were no significant differences (*p* > 0.05) in responses to heat stimulation between the uninjected control paw, and CFA-injected paw 7 days after 4R (15 mg/kg) treatment (Fig. 2f). In contrast, but also consistent with the male results (Fig. 1c-d), heat hypersensitivity was only partially reversed 2 days after 4R (15 mg/kg) systemic treatment and cold hypersensitivity was partially reversed at all time-points tested in females. Thus, responses to cold stimulation in the CFA-injected paw were significantly (*p *< 0.05) higher on days 2 and 7, and responses to heat stimulation were significantly lower on day 2, after 4R (15 mg/kg) treatment, when compared to the uninjected paw in vehicle condition at the same time-points (Fig. 2b-c, e). In both days tested, sensitivity to cold and heat stimulation of the uninjected hind paw was indistinguishable between vehicle and 4R treated groups.

Evaluation of CFA-induced tactile hypersensitivity in female mice showed that, unlike in males, withdrawal thresholds are comparable in the injected paws of 4R and vehicle-treated mice in both time points evaluated, demonstrating that tactile hypersensitivity is unaltered by 4R treatment (Fig. 2d, g). Similar to the male results, paw withdrawal thresholds in the uninjected hind paws are also indistinguishable in 4R and vehicle-treated female mice, demonstrating that baseline nociception and locomotor responses to peripheral stimuli are unaltered by 4R treatment. Altogether, these findings show that single systemic administration of 4R (15 mg/kg) reduces inflammation-induced thermal but not tactile hypersensitivity in female mice without altering baseline responses.

### 4R reduces inflammation-induced paw edema in male but not female mice

**Fig. 3:**
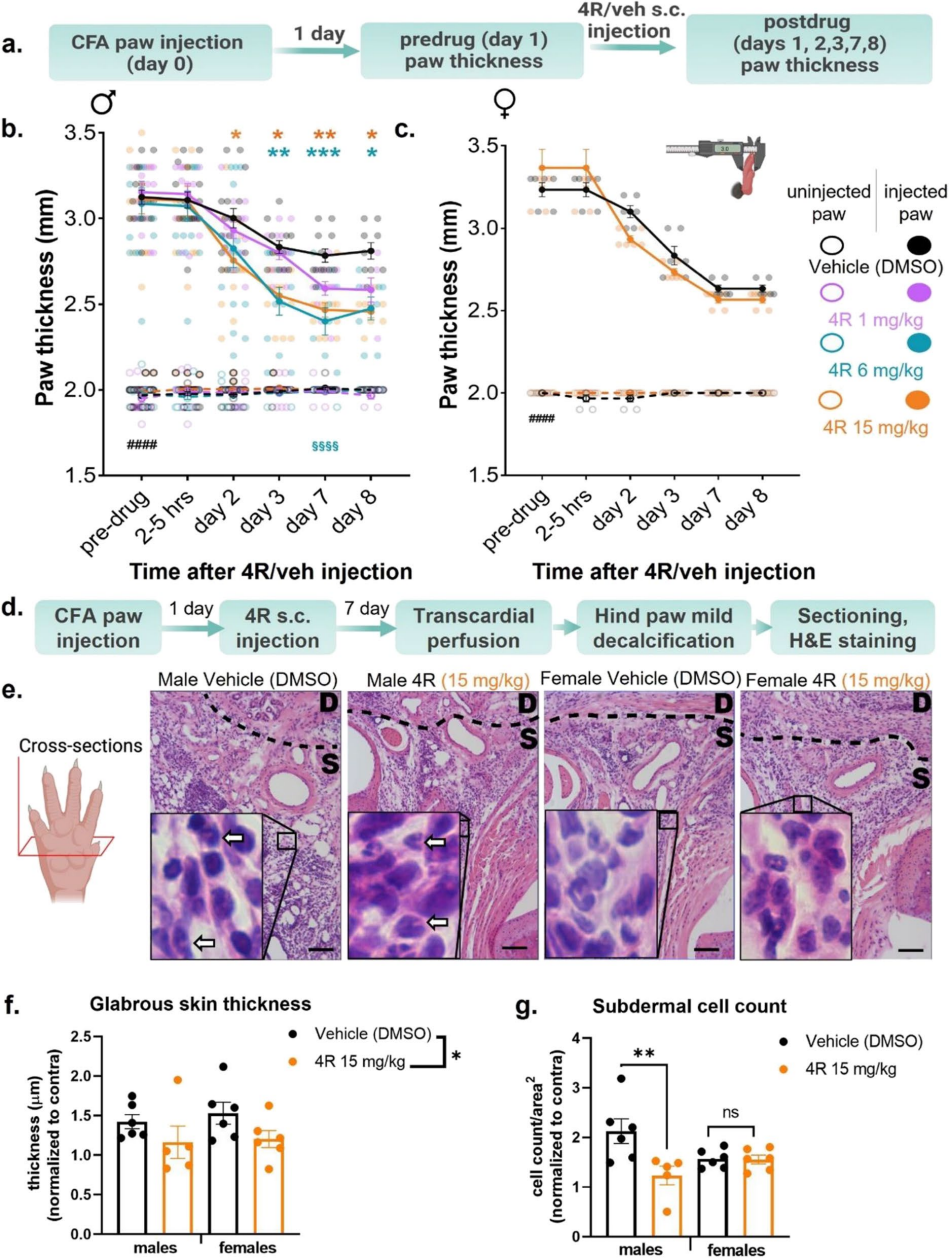
4R reduces inflammation-induced paw edema in male but not female mice (**a**) Experimental timeline for paw thickness measurements. In vivo thickness measurements of CFA-injected and non-injected hind paws before and 1–8 days after 4R (1, 6, and 15 mg/kg) or vehicle systemic administration of (**b**) male and (**c**) female mice. All values are expressed as mean ± SEM. *n* = 6–16 animals per treatment and sex. Two-way ANOVA (**b**) or Two-way RM ANOVA (**c**) followed by posthoc Tukey’s multiple comparisons test: CFA-injected paw vs non-injected paw in pre-drug vehicle (veh) conditions, *p* < 0.0001 (####); 4R CFA-injected paw vs non-injected vehicle conditions day 7, *p* < 0.0001 (§§§§), Two-way ANOVA followed by posthoc Dunnett’s multiple comparisons test: vehicle vs 4R systemic treatment in CFA-injected paws of the same time-point, *p* < 0.05 (*), *p* < 0.01 (**) and *p* < 0.001 (***). Results of multiple comparisons between vehicle (veh) and 4R 1 mg/kg, 6 mg/kg or 15 mg/kg are shown in purple, teal and orange asterisks, respectively. (**d**) Experimental timeline for histology samples. **e** Representative H&E images of dermal (D) and subdermal (S) skin, open arrows point to polymorphonuclear immune cell infiltration. Scale Bar: 200 µm. Histological analysis of hind paw skin 8 days post local CFA and 7 days post 4R (**f**) glabrous skin thickness of the hind paws and (**g**) subdermal immune cell count. All values are expressed as mean ± SEM. *n* = 5–6 mice per treatment and sex. Two-way ANOVA followed by Šidák’s multiple comparisons test: Vehicle vs 4R, *p* < 0.05 (*), *p* < 0.01 (**)

Previous studies have shown that systemic treatment with 4R has anti-inflammatory effects in the lipopolysaccharide model of inflammation in mice [61]. To test if the 4R anti-hyperalgesic effects described in the sections above are coupled to decreases in inflammation, we evaluated paw thickness as an indirect measurement of inflammation in CFA-injected and uninjected hind paws before and at different time points following the systemic administration of various doses of 4R (1, 6 or 15 mg/kg by body weight) or vehicle (Fig. 3a). As shown in Fig. 3b-c, subcutaneous injection of CFA in the hind paw of control male and female mice (systemically treated with vehicle) resulted in significant (*p* < 0.0001) increases in paw thickness (edema) when compared to their respective uninjected hind paws for the duration of the experiment. Evaluation of paw edema in male mice systemically treated with 4R revealed that a single administration of 4R attenuates inflammation-induced paw edema in a dose and time-dependent manner (Fig. 3b). Thus, male mice treated with the highest (15 mg/kg) and intermediate (6 mg/kg) doses of 4R showed significant (*p* < 0.05) reductions in paw thickness compared to vehicle-treated mice. 4R-mediated decreases in CFA-induced paw edema were first observed on day 2 after systemic 4R treatment and lasted for the duration of the experiment (8 days post 4R treatment). In contrast, paw thickness in CFA-injected hind paws of males treated with the lowest dose (1 mg/kg) of 4R was indistinguishable from those measured in vehicle-treated mice at all time points tested. Further analysis showed that paw thickness at the maximum anti-inflammatory effect, measured 7 days after a single dose of 4R (6 mg/kg), is significantly (*p* < 0.0001) higher in the CFA-injected paw, when compared to the uninjected control paw (Fig. 3b), demonstrating that 4R treatment partially reverses the inflammation caused by CFA injection. Importantly, paw thickness in uninjected hind paws were comparable in all treatments independently of dose or time-point, demonstrating that 4R-mediated decreases in paw thickness are specific to the inflamed hind paw.

Parallel experiments were performed to test if 4R-mediated decreases in inflammation-induced paw edema were also observed in female mice. Similar to male mice, female mice also developed paw edema after CFA injection that lasted for the duration of the experiment when compared to the uninjected hind paws (*p* < 0.0001) (Fig. 3c). In marked contrast to the results observed in male mice, however, measurement of CFA-induced paw edema in females after systemic treatment with 4R (15 mg/kg by body weight) were indistinguishable from those in females treated with the vehicle at all time points measured. These results demonstrate that systemic administration of 4R (15 mg/kg by body weight) reduces inflammation-induced paw edema in male but not female mice.

We used a histological approach to further investigate how systemic 4R treatment (15 mg/kg) influenced glabrous skin thickness after CFA injection on day 8 (7 days post-4R treatment) (Fig. 3d-e). Similar to previous studies, there is a robust increase in glabrous skin thickness at 8d post-CFA injection in male and female mice. However, our findings reveal that a single dose of 4R treatment resulted in a significant (*p* < 0.05) reduction in CFA-induced glabrous skin thickness independent of sex (Fig. 3f). In addition to the presence of edema, another hallmark feature of inflammation is a robust increase in immune cell infiltration into the local site of injury. We observed robust subdermal cell counts in the male CFA-injected group. Intervention with systemic 4R 1 day post-CFA injection led to a significant reduction in subdermal cell count in males suggesting an attenuation to the CFA-induced inflammation (Fig. 3g). Evaluating the effect of 4R on immune cell infiltration in female mice showed that the reduction in subdermal cell count occurred independently of 4R treatment revealing that there may be sex-driven differences in the way immune cells respond to 4R treatment (Fig. 3g).

### Pretreatment with an α7 nAChRs selective antagonist prevents 4R-induced reductions in inflammation-induced hypersensitivity and paw edema

**Fig. 4:**
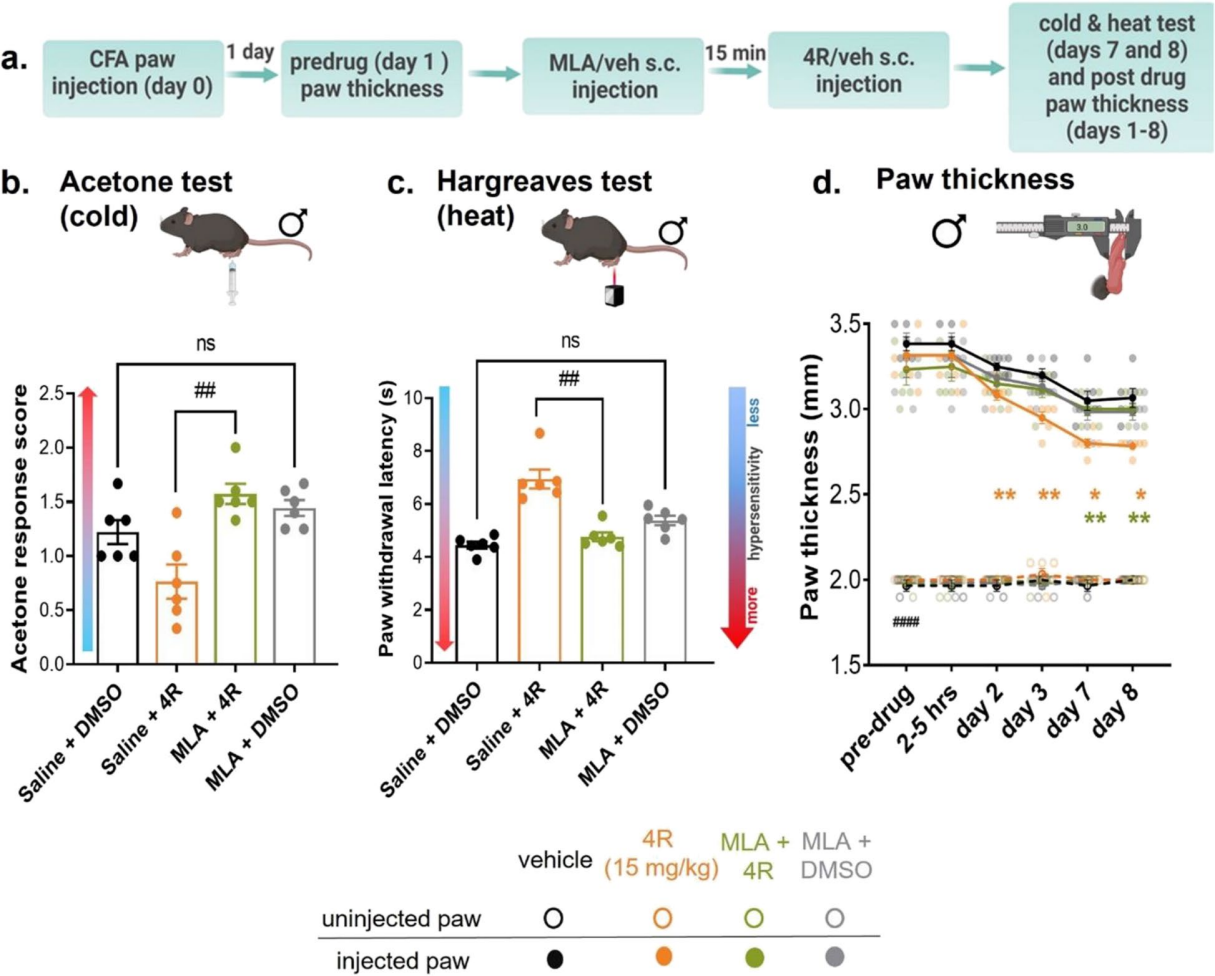
Pretreatment with an α7 nAChRs selective antagonist prevents 4R-induced reductions in inflammation-induced hypersensitivity and paw edema (**a**) Experimental timeline. CFA-injected and non-injected paw thickness measurements were taken in pre-drug condition. One day after CFA paw injection, MLA (10 mg/kg) or vehicle (veh) was systemically administered and 15 min after, animals received 4R (15 mg/kg) or veh treatment (s.c.). CFA-injected and non-injected paw thickness was measured 1–8 days after 4R (15 mg/kg), MLA (10 mg/kg) or vehicle administration. Acetone (cold) and Hargreaves (heat) tests in CFA-injected and non-injected hind paws were performed 7–8 days after systemic drug administration. (**b**, **c**) Acetone response scores (**b**) and paw withdrawal latencies in response to heat stimulation (**c**); Kruskal–Wallis test followed by posthoc Dunn’s multiple comparison test; saline + 4R vs. MLA + 4R; *p* < 0.01 (##); saline + DMSO vs. MLA + DMSO, *p* > 0.05, not significant (ns). (**d**) Thickness measurements of CFA-injected and non-injected hind paws; Two-way RM ANOVA followed by posthoc Tukey’s multiple comparisons test: CFA-injected paw vs uninjected paw in pre-drug veh conditions, *p* < 0.0001 (####). Two-way RM ANOVA followed by posthoc Tukey’s multiple comparisons test: CFA-injected paws in saline + DMSO vs saline + 4R systemic treatments and MLA + 4R vs saline + 4R at the same time-point, *p* < 0.05 (*), *p* < 0.01 (**). All values are expressed as mean ± SEM. *n* = 6 animals per treatment and test

Previous studies have shown that 4R modulates human nicotinic acetylcholine receptors (nAChRs) [15]. Separate studies have further identified nAChRs, particularly the α7 nAChRs, as potential pharmacological targets for the modulation of pain and inflammation [6, 62, 63]. Based on these combined findings, we hypothesized that the observed 4R-induced decreases in inflammation-induced hypersensitivity and paw edema in mice are mediated via modulation of α7 nAChRs. To test this hypothesis, the α7 nAChRs selective antagonist MLA (10 mg/kg, s.c.) was administered 15 min prior to 4R injection (15 mg/kg, s.c.) in male mice (Fig. 4a). CFA-induced cold and heat hypersensitivity were measured on days 7 and 8 after systemic injections, respectively, corresponding to the time points where we observed higher 4R-mediated anti-hyperalgesic and anti-inflammatory effects (Figs. 1, 2 and 3).

Consistent with the results presented in Fig. 1, animals treated with 4R (15 mg/kg) exhibited lower response scores to acetone stimulation and longer paw withdrawal latencies in response to heat stimulation of the CFA-injected paw compared to vehicle-treated animals, indicating that 4R reduces inflammation-induced cold and heat hypersensitivity (Fig. 4b-c). Evaluation of animals pre-treated with the α7 nAChRs selective antagonist MLA (10 mg/kg) revealed that MLA pretreatment prevents the anti-hyperalgesic effects of 4R (Fig. 4b-c). Consequently, mice systemically treated with MLA prior to 4R show significantly (*p* < 0.01) higher response scores to acetone stimulation (Fig. 4b) and significantly (*p* < 0.01) shorter paw withdrawal latencies in response to heat stimulation (Fig. 4c) of the CFA-injected paw compared with responses measured in animals pre-treated with MLA vehicle followed by 4R. Analysis of CFA-induced paw edema further revealed that, consistent with the results shown in Fig. 3b, a single injection of 4R (15 mg/kg) significantly (*p* < 0.05) reduced CFA-induced paw edema starting at day 2 post 4R treatment when compared to vehicle-injected male mice (Fig. 4d). Pretreatment with MLA (10 mg/kg) prevented the 4R-mediated reductions in paw thickness at days 7 and 8 after treatment. Importantly, CFA-induced thermal hypersensitivity and paw edema were not measurably affected by administration of MLA in the absence of 4R compared to vehicle-treated mice, demonstrating that systemic administration of the α7 nAChRs selective antagonist MLA does not measurably affect responses to cold or heat stimulation or CFA-induced paw edema (Fig. 4b-d). Collectively, these results demonstrate that 4R-induced reductions in inflammation-induced hypersensitivity and paw edema are mediated by positive modulation of ⍺7 nicotinic acetylcholine receptors.

### α7 nAChRs are expressed on a subset of subdermal macrophages but are not upregulated after CFA or 4R treatment

**Fig. 5:**
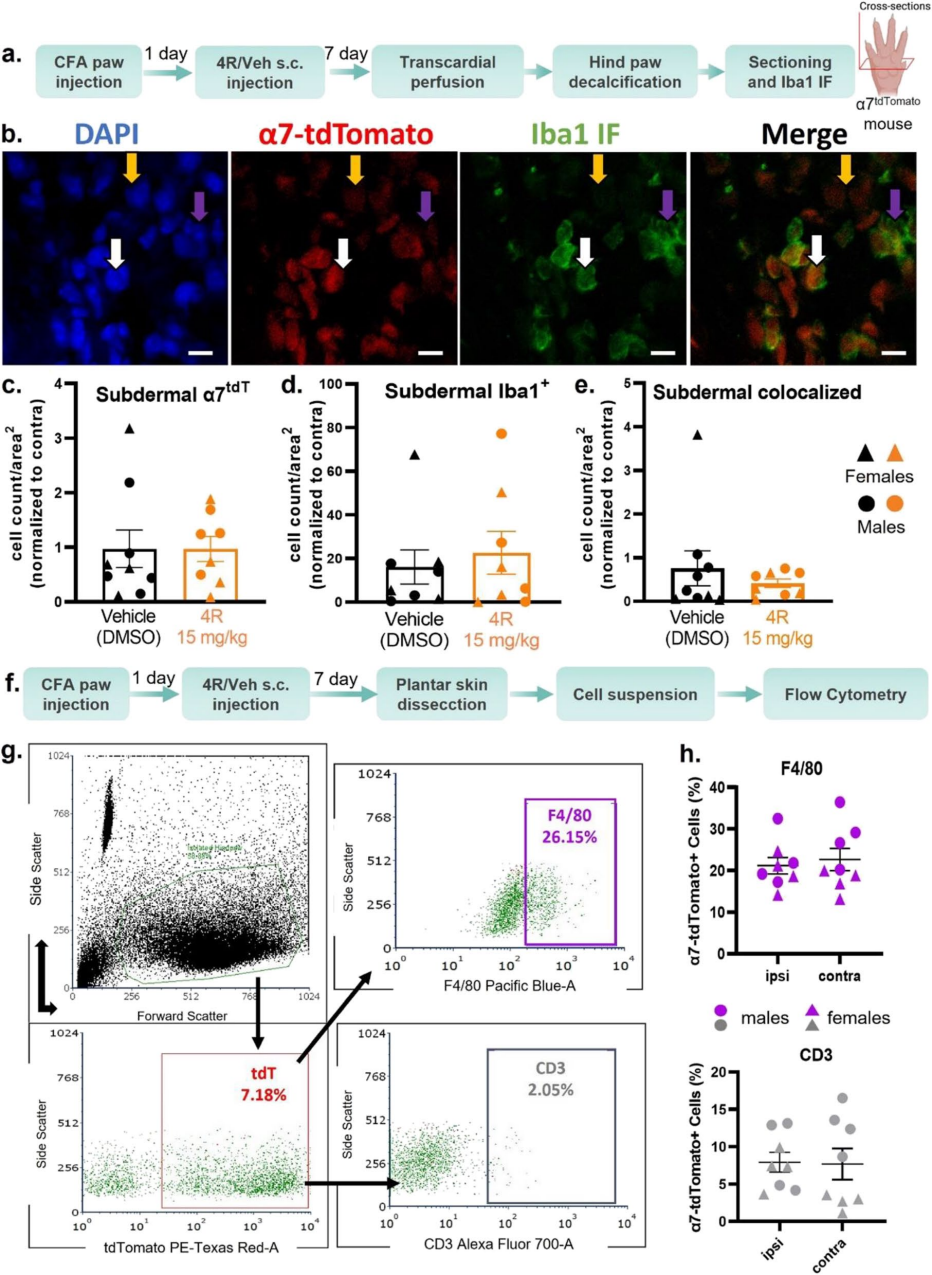
α7 nAChRs expression in hind paw macrophages (**a**) Experimental Timeline. Immunohistochemistry of α7^tdT^/Iba1^+^ macrophages in CFA-injected and non-injected hind paw skin (subdermal) 8 days post-CFA (day 0) and 7 days post 4R (15 mg/kg) or veh treatment (s.c.) (day 8). (**b**) A representative image of α7^tdT^ and Iba1^+^ hind paw subdermal co-expression. Scale bar: 20 µm. **c** Cell counts normalized to the area of subdermal α7^tdT^ cells. (**d**) Cell counts normalized to the area of subdermal Iba1 + cells. (**e**) Cell counts normalized of colocalized α7^tdT^ and Iba1^+^. (**f**) Experimental Timeline. Flow cytometric analysis of hind paw subdermal macrophages (F4/80) and T-cells (CD3) of CFA-injected (ipsi) and non-injected (contra) hind paw skin 8 days post-CFA only. (**g)** Hind paw skin was enzymatically dissociated and stained with F4/80, CD3, and endogenous α7^tdT^. After gating for dissociated hind paw skin cells based on forward and side scatter, cells were differentiated by their tdTomato^+^ signal, then subdivided into F4/80 and CD3 populations. (**h**) Analysis of overlapping of tdTomato^+^ cells that are either macrophages or T-cells in males and females

Due to the previously described role of macrophages in the innate immune responses [64–66], we hypothesized that α7 nAChRs ^+^ macrophages might play a role in analgesia and resolving inflammation and are modulated after 4R treatment in Iba1^+^ macrophages. However, when colocalizing α7^tdT+^ and Iba1^+^ cells, we found no significant changes to normalized cell count in the subdermal region of the hind paw at day 8 post-CFA (Fig. 5a-e). We also wanted to determine if α7^tdT^nAChRs overlapped with CD3^+^ T-cells and F4/80^+^ macrophages in whole skin and performed flow cytometry on dissociated hind paw skin from CFA-treated (ipsi) and non-treated (contra) male and female animals. Similar to the above IHC experiment, we did not observe a significant change in α7^tdT+^ cells after CFA but found roughly 7% of dissociated cells were α7^tdT+^ and close to 20% of these cells are F4/80^+^ macrophages and 10% are CD3^+^ T-cells (Fig. 5f-h).

## Discussion

The link between tobacco and pain has been known for decades [3]. However, its potential to treat pain is hindered by the harmful and highly addictive properties associated with tobacco use. The potential function of other components of the tobacco plant for pain treatment is understudied. In the present study, we investigated whether systemic administration of the 4R tobacco cembranoid results in anti-inflammatory effects and pain relief in a mouse model of inflammatory pain. Our findings demonstrate that systemic administration of 4R reduces peripheral hyperalgesia in both male and female mice. We further show that 4R reduces inflammation-induced paw edema and hind paw subdermal immune cell infiltration in males but not females, supporting the growing body of evidence demonstrating that modulation of pain and inflammatory responses is sexually dimorphic [57, 58, 60, 67]. Notably, our experiments demonstrate that 4R-mediated analgesia and anti-inflammatory effects last up to 8d after a single systemic administration, highlighting its potential therapeutical use in reducing persistent inflammation and pain. Lastly, using a pharmacological approach, our behavioral experiments show that 4R-mediated reductions in peripheral hyperalgesia and inflammation-induced paw edema are driven by the modulation of nicotinic receptors, particularly the ⍺7 nAChRs. Altogether, our findings identify the 4R tobacco cembranoid as an analgesic and anti-inflammatory agent and further support targeting the cholinergic system for pain and inflammation treatment.

### 4R-mediated anti-hyperalgesic and anti-inflammatory effects are sexually dimorphic

Our behavioral experiments using the CFA model of inflammatory pain show that a single systemic administration of 4R reduces inflammation-induced heat hypersensitivity in both male and female mice (Figs. 1d and 2c). However, we also show that 4R-mediated reductions in inflammation-induced tactile and cold hypersensitivity, albeit modest, are only observed in males but not females, despite the robust inflammation-induced hypersensitivity seen in both sexes (Figs. 1c, e and 2b, d). Similarly, systemic treatment with 4R results in reductions in inflammation-induced paw edema in males but has no effect in females despite the robust inflammation-induced paw edema elicited in both sexes (Fig. 3). Collectively, these results demonstrate that 4R-mediated anti-hyperalgesic and anti-inflammatory effects are sexually dimorphic, increasing the evidence in support of distinct mechanisms for inflammation and pain processing in males and females [57, 58, 60] and stressing the importance of incorporating both sexes in preclinical studies.

An important caveat of our studies is that we only evaluated one 4R dose (15 mg/kg) in females, whereas a full dose response was performed in male mice. We selected this dose for the female experiments because it consistently reduced inflammation-induced hyperalgesia and paw edema in males. This concern is mitigated by the similarities in the 4R-mediated anti-hyperalgesic effects observed in males and females. It suggests that the lack of measurable effect in paw edema in females is not due to the experimental dose selected. Given that previous studies evaluating the pharmacokinetics and metabolism of 4R in vivo have only been performed in male rats [45], future experiments to address potential sex differences in 4R drug activity are needed.

### Anti-hyperalgesic and anti-inflammatory effects after a single systemic dose of 4R are prolonged

**Fig. 6:**
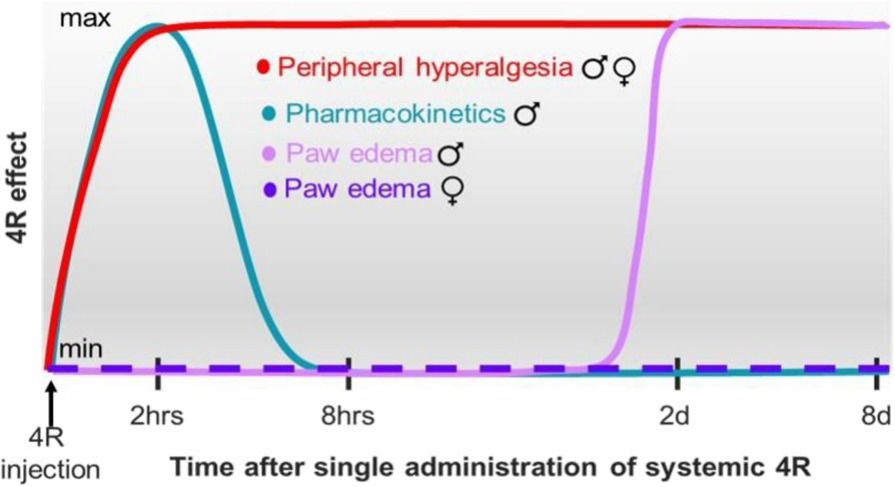
4R effects as a function of time. (**a**) Diagram of 4R effects in males and females as a function of time. Effects on peripheral hyperalgesia (red line) and paw edema (pink line for males and purple line for females) are shown relative to previously published pharmacokinetics [68] (blue line)

Previous pharmacokinetic and metabolic experiments have shown that the half-life of 4R, when administered systemically in rats, is between 36 min to 1.5 h [45]. These studies further showed no measurable plasma (or brain) levels of 4R 8 h post-administration. Consistent with the reported pharmacokinetics of 4R, our behavioral experiments show 4R-mediated anti-hyperalgesic effects 2.5 h after a single systemic 4R administration (Fig. 1). Notably, however, our behavioral time course further showed that 4R anti-hyperalgesic effects are long-lasting, with robust effects measured up to 8 days after administration of a single dose of 4R (Figs. 1 and 2). These results show that the behavioral effects of a single dose of 4R are persistent, outlasting the half-life of 4R, and present at time points when 4R is no longer measurably detected in the plasma or brain [45] (Fig. 6).

It is important to note that 4R effects are restricted to the inflamed paw, as no measurable effect was observed in the uninflamed paw. These results highlight that 4R treatment does not alter baseline sensory and locomotor responses to noxious stimulation, an evolutionary function essential for survival. These findings are also consistent with previous studies in rats that demonstrate that locomotor activity is not affected by systemic 4R administration [15]. A significant translational consideration in future studies is whether 4R produces dependence or addiction, as has been reported with other commonly used treatments for pain such as opioids [69]. This is particularly important given that 4R has been shown to bind to and modulate α4β2 nAChRs [15, 28], which have been previously linked to nicotine addiction [70, 71]. However, these concerns are mitigated by studies demonstrating that 4R reduces nicotine-induced withdrawal-like effects in planarians [72] and blocks behavioral sensitization to nicotine in rats [15]. Whether 4R treatment at the doses and time-points used in the present study are rewarding and potentially addictive, remains unknown and will be important to determine in the future.

At the inflammatory level, our results show that 4R-mediated decreases in inflammation-induced paw edema are delayed and not detected until 2 days post systemic 4R treatment (Fig. 3), when 4R is no longer detected in the body [45] (Fig. 6). Similar to the behavioral effects, the anti-inflammatory effects of a single dose of 4R are also persistent, with decreases in paw edema measured up to 8 days after administration of a single dose (Fig. 3). An important caveat of these experiments is that paw edema is an indirect gross measurement of inflammation and is limited by the sensitivity of the assay. Therefore, experiments to determine the effects of 4R in inflammatory responses at microscopic, cellular, and biochemical levels are warranted. Nonetheless, our findings demonstrate that 4R-mediated anti-inflammatory effects persist after the drug is eliminated from the body.

Altogether, our experiments show that 4R anti-inflammatory and anti-hyperalgesic effects outlast drug duration in the body (Fig. 6), suggesting that 4R effects occur via modulation of downstream signaling pathways. An alternative explanation is that a single dose of 4R administered shortly after injury interferes with the inflammatory response and subsequent development of persistent inflammation and hypersensitivity. Potential effects in central and/or peripheral nervous systems independent of the inflammatory response are also possible. Whether the anti-inflammatory and anti-hyperalgesic effects of 4R depend on the timing of drug administration relative to injury remains to be elucidated. Previous experiments have shown, for example, that systemic 4R treatment administered before and after brain injury in rats reduces neuronal death and inflammation [25]. Thus, it will be informative to know if systemic administration of 4R prior to injury also prevents the development of peripheral inflammation and hypersensitivity in mice, which could have important applications in preemptive analgesia. Determining the effects of 4R when administrated at later time points following injury will also be necessary. Lastly, longitudinal studies are also needed to determine if 4R treatment results in faster injury-induced inflammation and hyperalgesia recovery.

### 4R reduces pain and inflammation via modulation of ⍺7 nAChRs

Our experiments demonstrate that systemic administration of the tobacco cembranoid 4R reduces inflammation-induced hypersensitivity and paw edema (Figs. 1, 2 and 3). Using pharmacological approaches, we further show that pretreatment with MLA, a selective ⍺7 nAChRs antagonist, prevents 4R-mediated anti-hyperalgesic and anti-inflammatory effects (Fig. 4). Together, these results demonstrate that the anti-hyperalgesic and anti-inflammatory effects of 4R are mediated via positive modulation of α7 nAChRs. Our findings are consistent with a large body of literature demonstrating that positive modulation of α7 nAChRs is analgesic and anti-inflammatory [63]. Previous studies in animal pain models, for example, have shown that pharmacological modulation of these receptors has anti-inflammatory, anti-allodynic, and anti-hyperalgesic effects that have also been associated with the downregulation of cytokine levels and inflammatory pathways [48, 73, 74]. Moreover, past studies have demonstrated that α7 knockout mice display increases in hyperalgesia, paw edema, and allodynia compared to wild type mice in the CFA model of inflammatory pain, while baseline responses to noxious stimulation are comparable between α7 knockout and wild type mice [55]. These findings suggest that α7 nAChRs contribute to an endogenous analgesic tone during persistent inflammation.

### α7 nAChRs are expressed on a subset of subdermal macrophages but are not upregulated after CFA or 4R treatment

Our findings show that the systemic administration of 4R elicits a male-specific reduction of hind paw subdermal immune cells, which parallels the reduction of edema seen in males. This sexually dimorphic anti-inflammatory effect of 4R adds to increasing evidence of the existence of sex-specific mechanisms in pain and inflammation [75]. It does not appear that local macrophages play a role in the observable α7 nAChRs mediated anti-inflammatory effects. We were unable to observe a significant change of α7^tdTomato^/Iba1^+^ colocalized cell populations (Fig. 5a-e). This suggests there is another major cell population that is contributing to the attenuation of the CFA-induced inflammatory pain model after 4R treatment. It is known that other immune cells infiltrate the skin after CFA treatment. In addition to the expression on macrophages and T-cells, α7 nAChRs are also expressed on, B-cells, neutrophils, mast cells [34, 76, 77]. Future studies are needed to evaluate the contribution of other α7 nAChRs-expressing immune cell types in 4R modulation of pain-related behaviors and inflammation to further our understanding of cellular mechanisms.

An important factor to consider in the interpretation of our pharmacological experiments is that MLA has been previously shown to bind, albeit to a much lower affinity, to α9α10 nAChRs [78]. Given that the reported relative affinity of MLA is much higher for α7 nAChRs than for α9α10 nAChRs [78–81], it is most likely that the anti-hyperalgesic and anti-inflammatory effects we see here are mediated via modulation of α7 nAChRs.

The results from our in vivo pharmacological experiments showing that 4R effects are mediated via ⍺7 nAChRs, along with previous studies in vitro demonstrating that 4R can also bind α4β2, α3β4, and α1β1γδ nAChRs [15, 28], confirm that 4R can modulate multiple nicotinic receptors. It is important to note that, to the best of our knowledge, 4R is not known to bind to or directly modulate ⍺7 nAChRs. Future studies to evaluate whether 4R binds to and directly modulates ⍺7 nAChRs are needed. Studies have shown, however, that multiple cholinergic receptors are expressed in different tissues and cell types contributing to neuroimmune responses [82]. It is therefore possible that 4R modulates ⍺7 nAChRs via an indirect mechanism that does not require direct binding to the receptor. For example, 4R may bind to and modulate a different type of cholinergic receptor within the endogenous cholinergic anti-inflammatory pathway, which subsequently leads to indirect modulation of ⍺7 nAChRs, ultimately decreasing inflammation and behavioral hypersensitivity. Determination of the relative affinity of 4R to different nicotinic receptors and further characterization of the mechanism of action of 4R in distinct targets are essential. Nonetheless, the beneficial effects of 4R treatment presented here, along with the neuroprotective, anti-tumor, anti-inflammatory, and antimicrobial effects reported in previous studies [17, 19, 20, 23, 27, 28, 83] suggest that 4R can non-selectively activate tissue and pathological state-specific cell signaling cascades downstream from neural and/or immune nicotinic receptors, improving diverse pathological states, including pain.

### Supplementary Information

**Additional file 1. **Raw Data Table.

## Data Availability

All data in this study is available from the corresponding author.

## Abbreviations

4R: [(1S, 2E, 4R, 6R, 7E, 11E)-cembra-2,7,11-triene-4,6-diol]
CFA: Complete Freund’s Adjuvant
DMSO: Dimethyl sulfoxide
MLA: Methyllycaconitine
nAChRs: Nicotinic acetylcholine receptors
s.c.: Subcutaneous
α7 nAChRs: Alpha7 nicotinic acetylcholine receptors
IHC: Immunohistochemistry
NIH: National Institutes of Health
NMR: Nuclear Magnetic Resonance
TLC: Thin layer chromatography
PBS: Phosphate-buffered saline
4% PFA/PB: 4% Paraformaldehyde in phosphate-buffered saline solution
H&E: Hematoxylin and eosin
NGS: Normal goat serum
BSA: Bovine serum albumin
Iba1: Ionized calcium-binding adaptor molecule 1
α7^tdT^^+^: TdTomato^+^ cre driven nAChRs
S.E.M.: Standard error of the mean
